# Iron status of regular voluntary blood donors

**DOI:** 10.4103/0973-6247.39504

**Published:** 2008-01

**Authors:** Vilsu I. Mahida, Apksha Bhatti, Snehalata C. Gupte

**Affiliations:** *Surat Raktadan Kendra and Research Centre, Gopipura, Surat - 395 001, Gujarat, India*

**Keywords:** Depletion of iron stores, iron deficiency, regular voluntary donors

## Abstract

**Background:**

Our blood bank is a regional blood transfusion centre, which accepts blood only from voluntary donors.

**Aim:**

The aim is to study iron status of regular voluntary donors who donated their blood at least twice in a year.

**Materials and Methods:**

Prior to blood donation, blood samples of 220 male and 30 female voluntary donors were collected. Control included 100 each male and female healthy individuals in the 18- to 60-year age group, who never donated blood and did not have any chronic infection. In the study and control groups, about 10% subjects consumed non-vegetarian diet. After investigation, 85 males and 56 females having haemoglobin (Hb) levels above 12.5 g/dl were selected as controls. Donors were divided into ≤10, 11-20, 21-50 and >50 blood donation categories. Majority of the donors in >50 donation category donated blood four times in a year, whereas the remaining donors donated two to three times per year. Haematological parameters were measured on fully automatic haematology analyzer, serum iron and total iron-binding capacity (TIBC) by biochemical methods, ferritin using ELISA kits and transferrin using immunoturbidometry kits. Iron/TIBC ratio × 100 gave percentage of transferrin saturation value.

**Statistical Analysis:**

Statistical evaluation was done by mean, standard deviation, pair *t*-test, χ^2^ and anova (*F*-test).

**Results:**

Preliminary analysis revealed that there was no significant difference in the iron profile of vegetarian and non-vegetarian subjects or controls and the donors donating <20 times. Significant increase or decrease was observed in mean values of various haematological and iron parameters in donors who donated blood for >20 times (*P* < 0.001), compared to controls. Anaemia, iron deficiency and depletion of iron stores were more prevalent in female donors (*P* < 0.05) compared to males and especially in those male donors who donated their blood for more than 20 times.

**Conclusion:**

Regular voluntary blood donors should receive iron supplementation to prevent iron deficiency and depletion in iron stores.

## Introduction

After donation of 450 ml of blood, a male donor loses 242 ± 17 mg and a female 217 ± 11 mg of iron.[1] The loss is quickly made up by mobilizing the iron stores in the form of ferritin followed by replenishing the iron stores, if the diet is adequate. As iron stores decrease, iron absorption increases.[2]

Earlier study[3] in India has reported that 73.8% of professional blood donors are anaemic. Several reports[4–6] in the literature have shown that blood donation has significant influence on iron stores of voluntary blood donors.

Present study has determined the iron profile of regular voluntary blood donors to evaluate their iron status.

## Materials and Methods

Before starting the study, approval of the Institutional Ethics Committee was obtained. Informed consent was taken from the blood donors and control group for their willingness to participate in the study. Proformas were filled to get the information regarding age, sex, weight and dietary habits, past and present illness, infections, etc. Subjects receiving iron therapy were not included in this study. Blood donors were recruited as per the rules stipulated by the Drugs Controller of India. By medical interview and clinical examination, donors having chronic infections were excluded. Our donors belonged to a middle-class or higher middle-class group and consumed good diet. Prior to blood donation, regular voluntary donor's (a person who has donated blood at least twice in a year) blood samples were collected in plain and EDTA vials. Donors did not donate the blood if the haemoglobin (Hb) concentration was below 12.5 g/dl. The donors were classified into four groups as per number of previous blood donations, i.e. ≤ 10, 11-20, 21-50 and >50.

Control group consisted healthy individuals in the age group of 18-60 years, who did not have any acute or chronic infection and never donated blood. The blood was collected in camps organized with the help of community leaders for our other project on “Iron profile of the population”. After completion of laboratory studies, the individuals having <12.5 g/dl were excluded from the control group.

The blood samples of donors and controls were investigated for various haematological parameters on a fully automatic haematology analyzer (Nihon Kohden, Japan). Estimation of serum iron and total iron-binding capacity (TIBC) were carried out by Dipyridyl method.[7] Serum ferritin was estimated using Radim enzyme linked immunosorbent assay kit, whereas transferrin concentration was determined by immunoturbidometric method using DiaSys kit. Transferrin measurement was carried out only on 86 samples, because initially we did not include this parameter in the study. The transferrin saturation value was calculated by the following formula:

$$\%\text{ Transferrin saturation}=\frac{\text{Serum Iron (μl/dL)}}{\text{TIBC (μl/dL)}}\times 100$$

The mean and standard deviation (SD) values were calculated on microsoft excel. The pair *t*-test and χ^2^ were employed to evaluate statistical significance.[8] The *F*-value was calculated by anova test used for the comparison of multiple means with unequal sample sizes.[8] The *P*-value below 0.05 was considered significant.

## Results

The study includes 250 blood donors, 100 male and 100 female non-donors (controls). As the cut-off Hb level for selection of blood donors is 12.5 g/dl, for comparison, 85 males and 56 female non-donors having Hb ≥12.5 g/dl were used.

On average, donors in categories ≤10 and 11-20 were donating the blood twice a year, 21-50 category donors three to four times and majority of the >50 category donors donated their blood four times in a year. Two donors had given their blood for more than 100 times.

The mean age in controls and donors ranged between 28 and 44 years. As seen in Table 1, Hb, mean cell volume (MCV) and mean cell haemoglobin (MCH) values gradually decreased as number of blood donations increased. A significant correlation was observed in the 21-50 and >50 blood donation categories by pair *t*-test (*P* < 0.001). Red cell distribution width (RDW) showed direct correlation in male donors donating their blood for 21-50 and >50 times. *F*-values obtained by anova test showed a significant difference in mean values of all haematological parameters of male donors (*P* < 0.01).

**Table 1:** Mean ± SD values of age and haematological parameters

Sex	Number of donations	Total number	Age (years)	Hb (g/dl)	MCV (fl)	MCH (g/dl)	RDW (%)
Male	0	85	28.2 ± 9.4	14.5 ± 1.3	83 ± 6.7	28.5 ± 2.8	12.9 ± 0.73
	≤10	100	32.5 ± 12.6	14 ± 0.9	84.2 ± 5.9	28.2 ± 3	13.1 ± 0.8
	11-20	50	33.7 ± 8.2	13.9 ± 1.3	82 ± 8.7	28.8 ± 3.1	13.2 ± 1.0
	21-50	50	36.6 ± 8.4	13.2 ± 1.6*	77.8 ± 8.4*	26.7 ± 3.1*	14.4 ± 2.3*
	>50	20	44 ± 7.3	13 ± 1.6*	79.5 ± 6.8*	27.5 ± 3*	14.1 ± 2.32*
Female	0	56	33.7 ± 12.6	13.2 ± 0.7	88.7 ± 5.9	28.6 ± 2.1	13.2 ± 0.5
	≤10	30	33 ± 6.1	13 ± 1.0	86.2 ± 5.8	28.7 ± 3	13.2 ± 1.6

Anova test revealed significant *F*-values for Hb, MCV, MCH and RDW of male donors; *P* < 0.01. Hb - haemoglobin, MCV - mean cell volume, MCH - mean cell haemoglobin, RDW - red cell distribution width.

* Statistically significant increase or decrease by pair *t*-test, *P* < 0.001

Table 2 illustrates the mean ± SD values of various iron status parameters. Pair *t*-test comparing control with different blood donation categories in males showed significant variation for all iron status parameters in 21-50 blood donation category (*P* < 0.001). The comparison of control with >50 donation category showed a significant association with all parameters except transferrin. *F*-values calculated by anova test also showed a significant relationship in males for all iron status parameters except transferrin (*P* < 0.01).

**Table 2:** Mean ± SD values of iron status parameters

Sex	Number of donations	Total number	Serum iron (µg/dl)	TIBC (µg/dl)	Serum ferritin (mg/ml)	Serum transferrin (mg/dl)	Transferrin saturation (%)
Male	0	85	82.2 ± 22.9	344 ± 36.6	58.8 ± 45.1	270 ± 44.9 (10)	23.9 ± 4.6
	≤10	100	81 ± 28.7	346 ± 37	48.5 ± 35.1	325.5 ± 84.8 (15)	23.4 ± 5.9
	11-20	50	81.6 ± 20.7	351.5 ± 28.7	64.4 ± 51.4	325.6 ± 77.9 (18)	32.2 ± 4.1
	21-50	50	65.7 ± 10.2	379.3 ± 23*	39.5 ± 29.8*	304.6 ± 62.3 (23)*	17.3 ± 1.7*
	>50	20	60.2 ± 9.6*	382.2 ± 19.1*	22.5 ± 19*	378.4 ± 62.3 (10)	15.8 ± 7*
Female	0	56	64 ± 14	349 ± 37	39 ± 21	303.5 ± 41 (5)	22.3 ± 7.6
	≤10	30	69.2 ± 17.4	358 ± 30.3	42.8 ± 21.3	322.7 ± 28 (5)	19.2 ± 7.6

Figures in the parentheses indicate number of samples tested for transferrin. Anova test revealed significant *F*-values for all iron status parameters except transferrin; *P* < 0.01. TIBC - total iron-binding capacity.

* Statistically significant increase or decrease by pair *t*-test, *P* < 0.001

The iron deficiency was considered when serum iron was <60 µg/dl, TIBC >380 µg/dl and serum transferring >360 mg/dl. Transferrin saturation <16% and serum ferritin value below 15 ng/ml indicated depletion of iron stores.

Table 3 gives the incidence of anaemia, iron deficiency and iron stores depletion in donors. Anaemia, iron deficiency and iron stores depletion were more prevalent in >20 donation categories (*P* < 0.01) but a highly significant relationship is seen with >50 donations. Compared to the male donors donating blood ≤10 times, female donors of the same category more frequently had iron deficiency and reduced iron stores (*P* < 0.05). Statistical evaluation by χ^2^ test showed significant differences in values.

**Table 3:** Anaemia, iron deficiency and iron depletion in blood donors

Sex	Number of donations	Total number	Hb <12.5 g/dl	Serum iron ≤60 µl/dl	TIBC >380 µg/dl	Serum ferritin ≤15 mg/ml	Transferrin saturation <16%
			*n* (%)	*n* (%)	*n* (%)	*n* (%)	*n* (%)
Male	≤10	100	2 (3.0)	3 (3.0)	7 (7.0)	0 (0.0)	5 (5.0)
	11-20	50	6 (12.0)	5 (10.0)	7 (14.0)	0 (0.0)	8 (16.0)
	21-50	50	8 (16.0)	12 (24.0)	13 (26.0)	5 (10.0)	12 (24.0)
	>50	20	5 (25.0)	6 (30.0)	7 (35.0)	2 (10.0)	7 (35.0)
Σχ^2^, 3 df			13.48	18.44	14.87	16.10	15.20
*P*			<0.001	<0.001	<0.001	<0.001	<0.001
Female	≤10	30	8 (26.7)	9 (30.0)	7 (87.5)	2 (25.0)	5 (16.7)
Male versus female	≤10						
		χ^2^, 1 df	19.8	20.0	5.9	6.8	4.4
		*P*	<0.001	<0.001	<0.02	<0.01	<0.05

TIBC - total iron-binding capacity, df - degrees of freedom

## Discussion

It is known that anaemia is highly prevalent in India. The iron deficiency is mainly due to nutritional deficiency of iron, worm infection and blood loss. The control group, which represented Surat city population showed 15% anaemia in males and 44% in females. Because for selection of blood donors, Hb cut-off level is 12.5 g/dl for comparison control participants having ≥12.5 g/dl Hb were selected. Similarly, subjects having infections were not included in this study. Dietary habits have significant influence on iron levels. Only 10% of our voluntary donors and controls consumed non-vegetarian diet. However, our preliminary analysis (data not shown) suggested that there was no significant difference in the iron profile of vegetarians and non-vegetarians. The reason is majority of non-vegetarians consumed meat/fish only once or twice a week and some even less frequently.

Blood banks have a responsibility to protect blood donors and it is necessary to prevent anaemia in them. In the present study, 9.5% male and 26.7% female regular voluntary blood donors developed anaemia, defined as Hb below 12.5 g/dl. A study[9] conducted in Bangalore, South India, has reported incidence of anaemia as 2.4% in male and 19.7% in female donors. In our study, male donors who donated ≤10 times had 3% incidence of anaemia but those who donated blood >50 times had 25% incidence of anaemia. Thus, it is clear that anaemia develops as number of blood donations increase. Anaemia was present in eight (26.7%) out of 30 female donors. All the female participants of this study were in pre-menopausal age group; hence, they may have developed anaemia. Our donors mainly belonged to middle-class or higher middle-class groups and consumed good diet. Therefore, diet may not be responsible for anaemia in them.

It is known that frequent blood donations can cause depletion in iron stores. Published studies[4–6] have used different parameters to detect iron deficiency and iron depletion in blood donors. We employed the parameters such as serum iron, TIBC and transferrin for detection of iron deficiency and transferrin saturation and serum ferritin for iron depletion.

This study has shown the inverse relationship of serum iron, ferritin and transferrin saturation with number of blood donations. Significant reduction in the levels of these three parameters was observed in the donors donating their blood for multiple times. The iron deficiency was more frequently observed in donors donating their blood for >20 times. A study[10] from Iran has reported iron deficiency in 28% male donors who donated their blood about five to ten times in previous 3 years, while in first-time donors (0 donation), incidence was 2%. In females, they found 12.5% incidence of iron deficiency in 0 donation category and 77.8% in five to ten donation category.

Serum transferrin measured on 86 samples did not show significant relationship with iron deficiency. TIBC and serum transferrin levels are increased in iron deficiency but decreased in inflammations and infections.[11] In country like India where chronic infections are common, these parameters probably have limited value.

Several studies[4,5,12] have used serum ferritin concentration as an indicator of iron stores. A North Indian study[13] found ferritin levels below 15 mg/dl in 49% and 100% of male and female donors, respectively among donors donating their blood twice a year. In our study, ferritin concentration <15 mg/dl was more frequently observed in male donors donating their blood >20 times. A Malaysian study[14] observed gradual decrease in serum ferritin levels according to the number of donations.

## Conclusion

Significant anaemia and iron deficiency was observed in >50 category of regular voluntary blood donors. The present study recommends that regular voluntary blood donors should receive iron supplementation to prevent iron deficiency and iron stores depletions in them. There is also a need to educate regular voluntary blood donors about iron deficiency.
